# *J*‑Resolved Molecular Fingerprinting by Parahydrogen Hyperpolarized Low-Field NMR

**DOI:** 10.1021/jacs.5c22871

**Published:** 2026-04-30

**Authors:** Zefan Zhang, Igor Savukov, Christian Hilty

**Affiliations:** † Chemistry Department, 14736 Texas A&M University, College Station, Texas 77843, United States; ‡ 5112 Los Alamos National Laboratory, Los Alamos, New Mexico 87544, United States

## Abstract

A *J*-resolved spectroscopy that depends on homonuclear scalar coupling in the strong-coupling regime and heteronuclear coupling in the weak regime expands complex peak patterns to a second axis. Hyperpolarization by Signal Amplification by Reversible Exchange (SABRE) enables the spectroscopy at a low magnetic field of 0.82 mT. Overlapping peaks of molecules such as 3-fluoropyridine and 3,5-difluoropyridine are resolved. Density matrix simulations of the ^1^H and ^19^F spins indicate a strong dependence on the signs and values of the *J*-coupling constants, including the homonuclear couplings that are not directly observable. The best matching peak positions and intensities predict coupling constants, including couplings between chemically equivalent nuclear spins, ranging in magnitude from 0.4 to 9.0 Hz for the two molecules. Simulations of other spin systems show unique patterns for molecules containing ^1^H and ^19^F or ^13^C. The dependence of the *J*-resolved peak patterns on all coupling constants in a spin system presents a new modality for portable and inexpensive identification of molecules.

## Graphical abstract

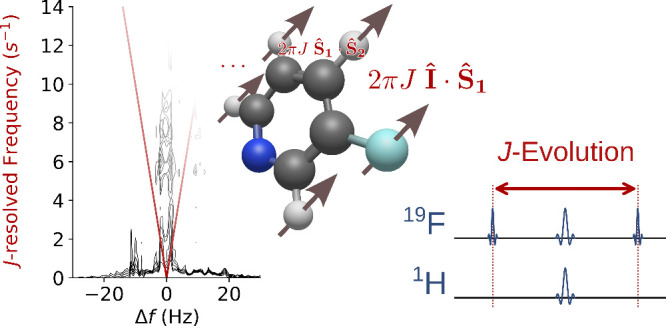

NMR spectroscopy derives much of its chemical specificity from the observation of chemical shift. Nevertheless, other parameters carry molecular information. The scalar coupling constants, also known as *J*-couplings, have long been used to predict dihedral torsion angles of biological macromolecules.^1^ Long-range couplings are sensitive to stereochemistry in addition to bond length and angle.^2,3^ Specific NMR experiments measure the magnitude and sign of *J*-couplings in molecules.^4,5^

*J*-couplings are informative at low magnetic fields, where chemical shifts cannot be measured. Low-field NMR requires less complex and costly instruments with increased portability.^6^ It also offers fundamentally new insights into molecular dynamics on an extended time scale,^7^ binding interactions,^8^ and chemical exchange.^9^ The line broadening caused by exchange is reduced or eliminated.^10^ The skin-depth at low RF frequency allows NMR observation through metal casings.^11,12^ Differing spin relaxation times provide alternative contrast in magnetic resonance imaging,^13^ and in differentiating the porosity of materials.^14^

*J*-couplings measured at low field identify structural motifs and enable the study of metabolism.^15,16^ Two-dimensional spectroscopy can correlate atom types and display coupling patterns even at Earth field.^17^ The high magnetic field homogeneity can improve the accuracy of *J*-coupling measurements.^18^ At a mT field, like spins fall into the strong-coupling regime, while heteronuclear couplings are weak compared to the frequency difference. Under strong coupling, the complexity of the spectra is increased.^19^ Coupling constants can be measured by spin-lock induced crossing and synchronized spin echoes^20,21^ or the combination of zero-field evolution with low-field detection.^22^ The *J*-evolution can further be directly observed in zero-field.^23,24^ Long-lived spin states exhibit unique features at low- and zero-field, whereby relaxometry properties are linked to *J*-coupling.^25,26^

Because of the low spin polarization, limited sample amounts for chemical analysis may not be detectable.^27^ Hyperpolarization enables measurements by enhancing signals 10^6^-fold or more in a mT field.^28^ Signal Amplification by Reversible Exchange (SABRE)^29^ is suitable for this purpose, allowing for repeatable polarization without reliance on bulky and expensive instruments.^30,31^

We aim to demonstrate molecular fingerprinting by *J*-resolved, two-dimensional NMR spectroscopy. The use of *J*-couplings at low field contrasts with chemical shift-based fingerprinting at high field.^32,33^ Pyridine derivatives, which are readily SABRE hyperpolarizable and are highly abundant motifs in biological molecules and pharmaceuticals,^34^ are chosen as model compounds.

Hyperpolarized NMR spectra of 3-fluoropyridine and 3,5-difluoropyridine, measured at 0.82 mT,^6,35^ are shown in Figure 1. Inhomogeneous broadening is refocused in a single spin–echo train, allowing data acquisition for 3.4 s. The frequency separation of ^19^F and ^1^H spins is too wide for precise excitation by a rectangular radio frequency (RF) pulse, prompting the use of two simultaneous selective pulses (Figures S3–S5).

**Figure 1:**
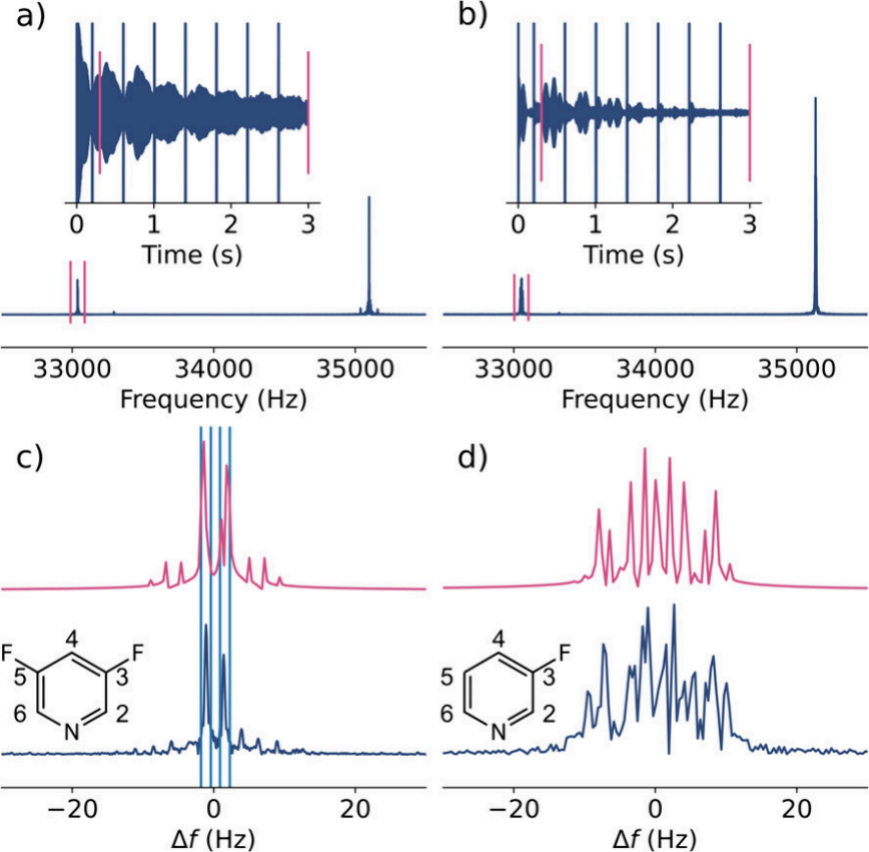
1D unblanked time-domain echo train and spectra of SABRE hyperpolarized (a) 3,5-difluoropyridine and (b) 3-fluoropyridine measured at 0.82 mT. The ranges for FT and magnification are indicated by magenta bounds. The time-domain signals are shown with a digital filter selecting the ^19^F Larmor frequency. (c,d) Corresponding experimental (blue; bottom) and simulated (magenta) ^19^F spectra processed with 4× zero filling, displayed as shifts from the ^19^F frequency. *B*_0_ and *B*_1_ inhomogeneity, *B*_1_ miscalibration and relaxation were not included in the simulations. Integration ranges are indicated (see text).

After blanking transient interference from the pulses, Fourier transforms (FT) were performed over all echoes. The *J*-splittings become resolved due to the application of pulses on both nuclei, refocusing inhomogeneous broadening but allowing the coupling-modulated coherences to evolve. The complex and overlapping peak patterns do not conform to high-field multiplet rules and can only be understood by considering the evolution of the spin system.

Density matrix simulations of all coupled ^1^H and ^19^F spins were performed using a set of reported coupling constants (eqs S1–S9).^36,37^ The peak positions in the simulations match the experimental spectra, providing validation of the protocol (Figures 1c,d). An asymmetry in signal intensity may be attributed to digital resolution and pulse excitation effects. The integrals of the two major peaks of 3,5-difluoropyridine differ by 8% in the experiment and are identical in the simulation. Other intensity differences are in part because the simulation did not include spin relaxation and field inhomogeneity effects.

The experiment in Figure 2 is designed to disperse the *J*-coupling information into a second dimension, reducing the peak overlap. Multiple echoes were recorded after the incremented *t*_1_ period (Figure 3). RF pulse interference remaining after phase cycling was digitally blanked. The coherence evolution during *t*_1_ is visible in the successive one-dimensional spectra.

**Figure 2:**
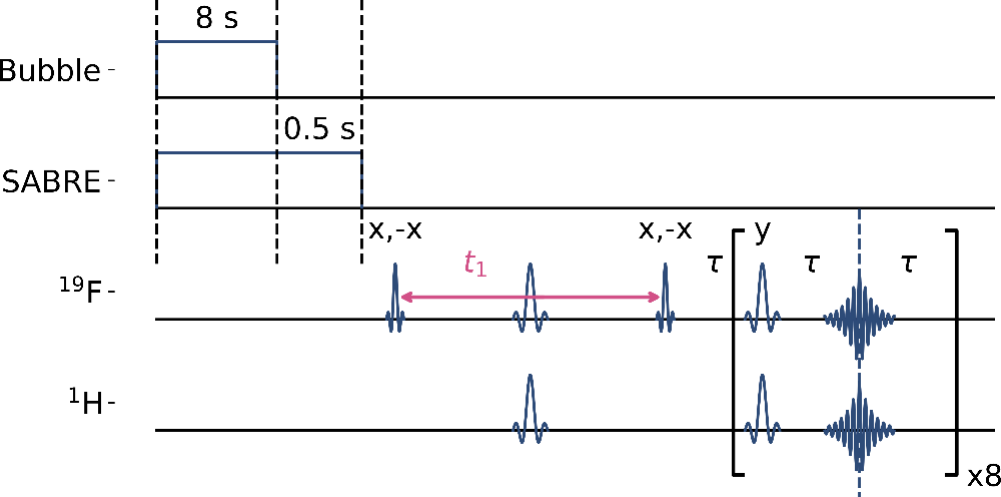
2D pulse sequence used in the low-field NMR experiment. Parahydrogen is bubbled while applying a magnetic field for SABRE polarization. The sinc shapes show π/2 and π pulses with *x* phase, unless otherwise indicated. *t*_1_ is the incremented *J*-evolution time. The time period covering the repeated echoes, each separated by 2*τ*, is processed by FT.

**Figure 3:**
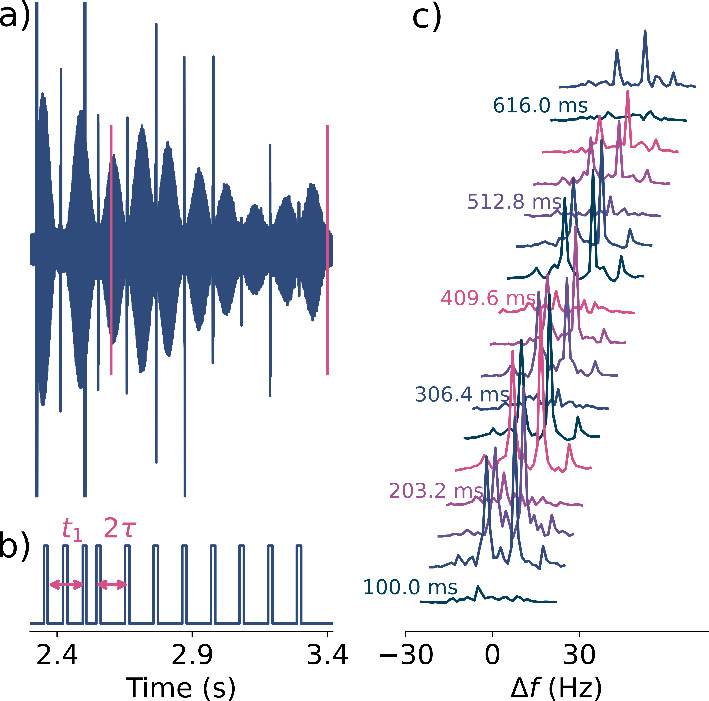
(a) Unblanked signal of the second *t*_1_ increment measured of SABRE hyperpolarized 3,5-difluoropyridine at 0.82 mT. The FT range is indicated by the magenta bounds. (b) Blanking range with marked evolution time and first echo time period. (c) 1D spectra of 3,5-difluoropyridine for the first 16 *t*_1_ increments of the 2D NMR experiment.

The 2D NMR spectra were obtained from the FT of the *t*_1_ dimension across all scans. The spectral region around the ^19^F Larmor frequency (Figure 4, Figure S8) contains only signals from the molecules of interest, omitting ^1^H signal from solvent or orthohydrogen produced during SABRE. The 3,5-difluoropyridine shows four peaks in a symmetrical rectangular pattern with 9.6 Hz spacing on the direct axis, positioned at 4.54 and 9.53 Hz on the indirect axis (Figure 4a). In contrast, the 3-fluoropyridine spectrum shows peaks in two columns symmetrically spaced 2.6 Hz apart on the direct axis, positioned at 1.81, 5.89, 7.45, 8.55, 10.45 and near 0 Hz (Figure 4b). A signal at zero frequency in the indirect dimension is due to the FT applied to real-valued magnitude spectra. Noise in the indirect dimension is attributed to the scan-to-scan signal fluctuation.

**Figure 4:**
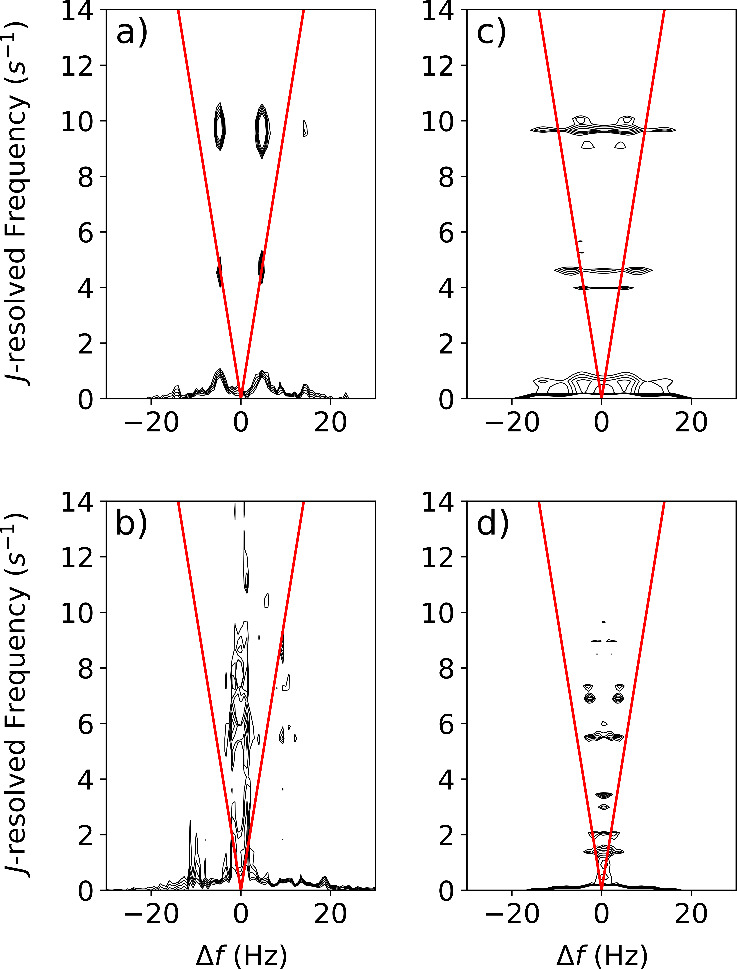
Two-dimensional *J*-resolved spectra of ^19^F in (a) 5 mM 3,5-difluoropyridine and (b) 5 mM 3-fluoropyridine measured at 0.82 mT with SABRE hyperpolarization. Simulated spectra of (c) 3,5-difluoropyridine and (d) 3-fluoropyridine with parameters matching the experiment. All ^1^H and ^19^F spins were simulated. Intensities are on a logarithmic scale with ceiling at maximum signal above 4 Hz on the direct axis, and floor at *e* ^–1^ of ceiling. The red “V” shapes indicate the positions for weakly coupled spin systems.

Product operator calculations illustrate the behavior of the pulse sequence in the weak coupling regime (eqs S10–S14). A sinusoidal modulation of observable in-phase coherences with respect to *t*_1_ evolves from the selected anti-phase coherences. The signals of two heteronuclear-coupled spins would fall onto a unique “V” shape in the 2D spectrum. In Figure 5, the absence of homonuclear coupling categorizes the system as weakly coupled, allowing the product operator calculation to predict the result. The slope of 2­(*J*_1_ + *J*_2_) in the V shape and the zero-frequency signal arise from the FT of magnitude values, which was performed in the same way as for the experimental data. Spectra processed without calculating the magnitude are shown in Figure S6.

**Figure 5:**
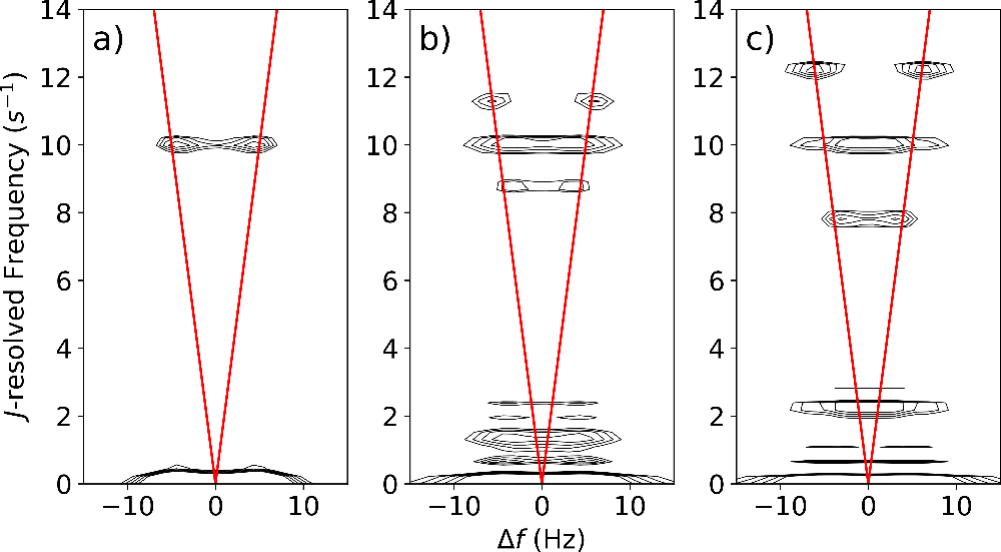
Simulated spectra for a three-spin system (I, S_1_, S_2_) at 0.82 mT, where *J*_IS1_ = 5 Hz, *J*_S1S2_ = 0 and (a) *J*_IS2_ = 0, (b) *J*_IS2_ = 0.5 and (c) *J*_IS2_ = 1 Hz. The signal of the I spin is shown. The red “V” shapes are predicted by product operators.

The signal patterns for both molecules deviate from the “V” shape (Figure 4). At 0.82 mT, the homonuclear couplings make the spin system fall into the strong coupling regime, which is outside the scope of the product operator formalism. The patterns resulting from the multiple couplings among the ^1^H and ^19^F spins are not readily predicted analytically. They act as unique fingerprints identifying each molecule resolved in the *J*-dimension.

The 2D spectra were simulated using numerical calculations of the density matrix evolution (Figures 4b,d). The couplings were illustratively determined by matching the patterns in simulated and experimental spectra. Starting with coupling constants from the literature,^36,37^ the signed values were manually adjusted to reproduce the positions and intensities of the strongest peaks. The simulated 2D spectral peak patterns strongly depend on the signs and values of coupling constants (Figure S10). The errors in the matched values were estimated by calculating the positional partial derivative of the strongest peak to the *J*-coupling value (Figure S11).

The final values representing the matched spectra remained in agreement with the starting values within 0.25 Hz (1.5-fold of estimated error values), with two exceptions: First, the ^19^F homonuclear coupling constant, *J*_35_, of 3,5-difluoropyridine was matched to a nonzero value at 4.76 Hz. Being chemically but not magnetically equivalent, the two ^19^F spins on the 3,5-difluoropyridine are allowed to form a scalar *J*-coupling interaction, which is not observable in one-dimensional spectra in the literature.^37^ Second, the ^1^H–^1^H *J*_*25*_, *J*_*26*_ and *J*_*36*_ couplings of 3,5-difluoropyridine, reported as zero, were found to have little impact on the simulated spectra and could not be resolved, possibly due to a doubly indirect role played in the evolution of ^19^F spins.

Because not all literature values reflect the signs of coupling constants, high-field NMR experiments were performed to independently obtain this information (Figures S14–S18). The simulation resulted in the same sign as the experimental results in all cases. As illustrated, the density matrix simulation further can indirectly determine the coupling constants between chemically equivalent spins.

Predicting the behavior of strongly coupled spin systems in many cases requires simulations.^38^ Here, the analysis is facilitated by the *J*-resolved frequency dimension. The peak positions resulting from *t*_1_ evolution depend sensitively on the coupling values, can be tracked to estimate error ranges in identified coupling constants and can be compared to the case of weak couplings (cf. “V”-shapes). This behavior contrasts to other methods for *J*-coupling determination at low field, SLIC and synchronized spin echoes, which manipulate level-anticrossing.^20,21^

The experiment deterministically transforms the scalar coupling constants into a complex pattern, thought of as an NMR “hash function” for molecular analysis. Simulations of the other stable mono- and difluoropyridines yielded unique fingerprints (Figure S19).^36,39^ The 2-fluoropyridine shows a vertical pattern at zero frequency; 2,3- and 2,6-difluoropyridines show two horizontally symmetrical dominating peaks at different positions. The 2,5-difluoropyridine shows four matrix-like peaks near 6 Hz on the indirect axis.

Molecules containing other NMR active isotopes such as ^31^P, ^13^C or ^15^N are amenable to pattern matching. At low field, any spin can be excited by changing the frequency of the RF pulse without instrumental modification.^40^ These options are exemplified with simulations of the metabolites pyruvic acid and lactic acid, which are readily SABRE hyperpolarized.^41,42^ The 1- and 2-^13^C labeled forms of the molecules are identified by their distinct fingerprints (Figure S20). A simulation with reduced *J*-resolution suggests that patterns could be measured in less than a minute (Figure S21). The fingerprinting efficiency in mixtures containing multiple types of nuclei may further be improved by Hadamard spectroscopy.^43^ Density matrix simulations could be accelerated by trained artificial neural networks.^44^

Fluoropyridines can be detected with high sensitivity using benchtop NMR with a permanent magnet in the tesla range.^45,46^ In contrast, the milli-tesla magnetic field is produced by an electromagnet alone (Figure S1), which may be developed into an inexpensive, portable device. Applications in the field include industrial process control, security, biomedical identification, and others. The method likely works best with some prior knowledge of the expected types of molecules, included in a database of patterns. Parahydrogen is compatible with remote applications, as it can be produced inexpensively by cooling hydrogen gas and stored for an extended time. One limiting factor is the nonuniformity of SABRE polarization, requiring a binding site for the polarization catalyst. In some cases, selective polarization can simplify observed patterns. In other cases, hyperpolarization in the molecules and spins of interest may be generated by relayed methods and heteronuclear polarization transfer.^47,48^

In summary, through a *J*-resolved 2D NMR experiment and SABRE signal enhancement, the coupling constants of 3-fluoropyridine and 3,5-difluoropyridine were compared and found in agreement to reported experimental values. Observed spectra and density-matrix simulations show distinct patterns sensitive to the signs and values of *J*-coupling constants. With the spectral fingerprint, the presence of known molecules, or potentially the structure of unknown molecules may be determined using low-cost, low-field NMR.

## Supplementary Material



## Notes

Experimental methods, low-field NMR calibration data, density matrix simulation of 1D experiments, product operator description of 2D experiments, validation of 2D spectra, density matrix simulation of 2D experiments, measurement of scalar coupling constants by high-field NMR, and simulation of molecules with expanded scope (PDF)

## Abbreviations

NMR: Nuclear Magnetic Resonance
RF: Radio Frequency
SABRE: Signal Amplification by Reversible Exchange
2D: Two-dimensional
