# Supplementary material for: J‑Resolved Molecular Fingerprinting by Parahydrogen Hyperpolarized Low-Field NMR
Source: J Am Chem Soc. 2026 Apr 30;148(18):18568–72. doi: 10.1021/jacs.5c22871 (PMC13184985; doi:10.1021/jacs.5c22871)
Supplement: Supplementary file 1 [file ja5c22871_si_001.pdf]

Supporting Information for

J-Resolved Molecular Fingerprinting by Parahydrogen Hyperpolarized Low-Field NMR

Zefan Zhang[†], Igor Savukov[‡] and Christian Hilty[†,*]
[†]Chemistry Department, Texas A&M University, College Station, TX 77843, United States
[‡]Los Alamos National Laboratory, Los Alamos, NM 87544, United States

[*]e-mail: chilty@tamu.edu

Table of Contents

1. Experimental Methods.......S2
  1.1 Sample Preparation.......S2
  1.2 Low-Field NMR Spectroscopy using SABRE.......S2
  1.3 Data Processing.......S4
  1.4. Density Matrix Simulations.......S4
  1.5 High-Field NMR Experiments for Measurement of *J*-Coupling Constants.......S6
2. Low-field NMR Calibration Data.......S7
3. Density Matrix Simulation of 1D Experiments.......S8
4. Product Operator Description of 2D Experiments.......S8
5. Validation of 2D Spectra.......S11
6. Density Matrix Simulation of 2D Experiments.......S13
7. Measurement of Scalar Coupling Constants by High-Field NMR.......S16
8. Simulation of Molecules with Expanded Scope.......S20
9. References.......S22

1. Experimental Methods

1.1 Sample Preparation

The SABRE active sample was prepared by the dispersing 5 mM of chloro(1,5-cyclooctadiene)[4,5-dimethyl-1,3-bis(2,4,6-trimethylphenyl)imidazol-2-ylidene] iridium(I) (Strem Chemicals Inc., Newburyport, MA), 50 mM of 3-fluoropyridine or 3,5-difluoropyridine (Ambeed Inc., Arlington Hts, IL) and 50 mM of D6-dimethyl sulfoxide (99.9% deuterated, Cambridge Isotope Laboratories, Inc., Andover, MA) into methanol (VWR International, Radnor, PA). The prepared samples were transparent orange liquids.

The parahydrogen gas was enriched from 5.0 UH-K hydrogen gas (Linde US, Danbury, CT) in a DE-204S cryocooler (Advanced Research Systems, Macungie, PA) working at 29 K, at 8.3 bar.

1.2 Low-Field NMR Spectroscopy using SABRE

The NMR experiments took place in a low-field NMR spectrometer as reported previously.[1,2] The constant magnetic field of 0.82 mT was generated by a tetracoil,[3] and a PCIe-6363 data acquisition board (National Instrument, Austin, TX) was used to simultaneously generate NMR pulses and acquire NMR signals (*Figure S1*). The voltages of RF pulses were amplified by a OPA4131PJ amplifier (Texas Instruments, Dallas, TX) with a gain of 2 before irradiation. The radio frequency (RF) detection coil was tuned to the Larmor frequency of ^{19}F. The orthogonal excitation coil was left untuned. Two Golay coils and one anti-Helmholtz coil, independent and mutually perpendicular, provided first order shimming on x, y and z axes. During the SABRE-NMR experiment, aliquots of 1.2 mL were loaded into a 10 mm NMR tube, connected to the parahydrogen delivery system and pressurized. The sample was then saturated with parahydrogen by bubbling for 5 minutes at 0.1 standard liter per minute (SLPM). During this time, the sample was observed to become a colorless transparent liquid.

The one-dimensional (1D) NMR spectra were measured using a Carr-Purcell-Meiboom-Gill (CPMG) experiment (*Figure S2*).[4] The echo time was 2τ, with $\tau = 0.2$ s. All radio-frequency pulses were $sin(x)/x$ shaped, with x extending from -3π to 3π, for separate selective excitation of ^{1}H and ^{19}F. The Larmor frequencies of ^{1}H and ^{19}F are determined as 35090 and 33020 Hz respectively. The $\pi/2$ pulse length of the ^{1}H and ^{19}F on-resonance shaped pulses were measured to be 3.01 and 3.38 ms respectively at the same maximum voltage amplitude at a generated 0.35 V amplitude before sum, as calibration of pulse length. The waveform for the simultaneous pulses was constructed as the sum of the individual pulse functions, centered in time. The CPMG spectra were averaged over 16 scans.

Parahydrogen was bubbled into the sample for 8 seconds at a flow rate of 0.1 SLPM parahydrogen gas before each scan. During and extending 0.5 s past this time, a 6.4 mT magnetic field was applied to the sample. A 0.6 second pre-scan delay followed the termination of the 6.4 mT magnetic field, after which the NMR pulse sequence commenced to avoid the effects of transient field. The signal was acquired during the entire pulse sequence after the pre-scan delay, including during the periods covered by the pulses. The sampling rate was 800,000 samples per second.

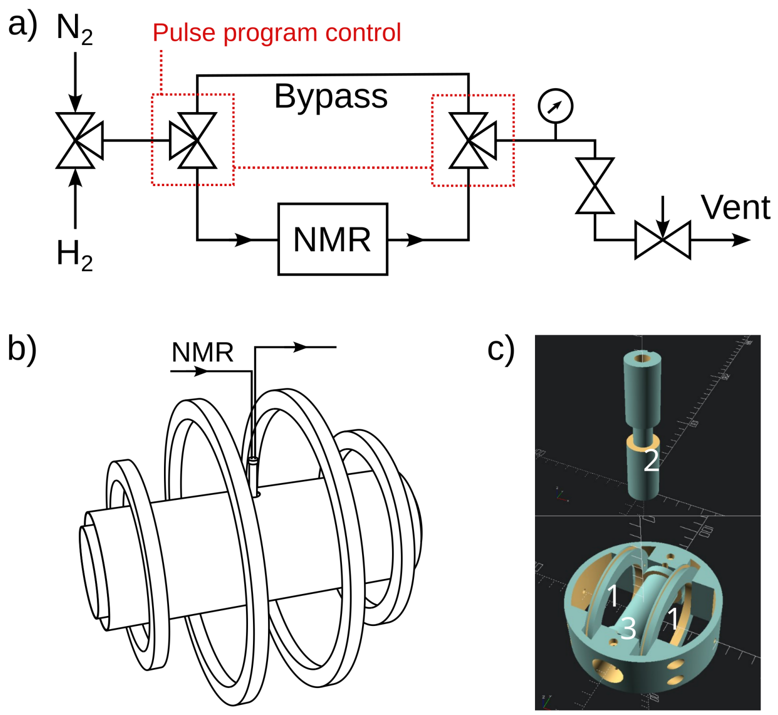

Figure S1: Instrumentation for low-field NMR spectroscopy. a) Flow path of parahydrogen. Valves are, from left to right, selection of N_2 purge gas or parahydrogen; dual valve for selection of bypassing or bubbling parahydrogen in NMR; shutoff valve for pressurization; needle valve for flow control. The bypass valves are controlled by the NMR pulse program. A pressure gauge is located after the bypass. b) Illustration of the NMR instrument (not to scale). The tetracoil comprises four solenoid rings. The inside cylinder is a cylindrical noise shield. The Golay coils for shimming are wound on a separate cylindrical form covering the shield. A sample tube is penetrating the shield at the top. The parahydrogen inlet and outlet are shown with arrows. The length of the instrument is approximately 50 cm. c) Rendering of NMR coil forms located inside of the cylindrical shield from (b). Bottom: Coil form comprising a Helmholtz coil for NMR pulses (1) and the a solenoid for field stepping to provide SABRE (3). Top: Form comprising solenoid for NMR detection, to be inserted in the form at the bottom.

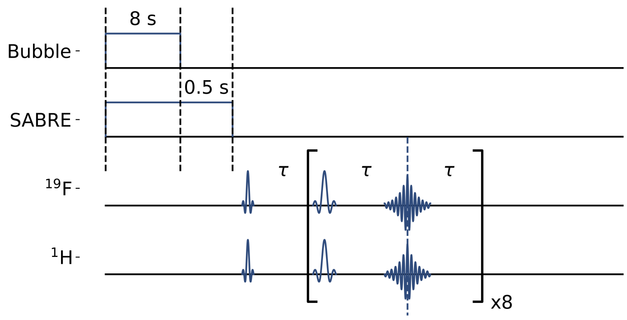

Figure S2: Pulse sequence for the one-dimensional low-field CPMG experiment. The narrow and wide sinc shapes denote π/2 and π pulses with corresponding envelopes, respectively. The oscillating curves denote the positions of the spin echoes. The time domain signal is Fourier transformed for the time period starting 0.3 s and extending to the end of the signal acquisition.

The two-dimensional (2D) NMR pulse sequence included an initial excitation and evolution phase, followed by a CPMG pulse train (*Figure 2*). The parameters were the same as for the 1D experiment, with the modifications described in the following. The evolution of *J*-couplings occurred during the t_1 time flanked by two ^{19}F π/2 pulses. The t_1 time was incremented from 0.1 s to 2.2672 s in steps of 0.0344 s for a total of 64 steps. To account for the signal loss to relaxation in the t_1 time, the last π/2 starts at 2.5 s for all increments. The two ^{19}F π/2 pulses were phase cycled by *x* and -*x*, followed by subtraction of the respective FID to construct a single FID corresponding to this t_1 time. The CPMG pulse train consisted of 8 refocusing pulses spaced by an echo time of 2*τ*, with *τ* = 0.05 s. The *τ* is shorter than in the 1D experiments because the signal observable time is reduced by the relaxation during t_1 time.

1.3 Data Processing

The NMR data were processed using Python (Python Software Foundation, www.python.org). The data was digitally blanked (zeroed) before and after 2 ms of each pulse to remove transient interference from the pulses. A Fourier transform was applied on the blanked real-valued data to produce the one-dimensional spectra, or the direct dimension of the two-dimensional spectra. Fourier transform was applied from 0.3 s to the end of the FID in 1D experiments, and from 2.5 s to the end of the FID in 2D experiments.

In the two-dimensional spectra, the indirect dimension was computed as the real Fourier transform of the direct dimension magnitude spectra over the incremented t_1 time. The magnitude spectra is the magnitude of the complex Fourier transform results of real FID. The 2D spectra were reported in logarithmic scale.

In Figure 1, the time domain signals were visually improved by applying a 4th order Butterworth digital filter between -1 and +1 kHz centered on the ^{19}F Larmor frequency to remove noise and unwanted NMR signal. No digital filtering was applied in any Fourier transformed spectra.

1.4. Density Matrix Simulations

Two-dimensional (2D) NMR spectra were simulated by numerical calculation of the density matrix in which the matrix represents the statistical ensemble of the coherences. The number of nuclear spins in the NMR experiment was large enough to be considered an ensemble whose observables could be ascertained by the average response from all wavefunctions, and the knowledge of each wavefunction was not necessary.[5]

A few assumptions were made in the simulation: First, Zeeman and scalar coupling Hamiltonian terms were considered only.

$$\hat{H}=\omega_{I_1} I_{1Z}+\omega_{I_2} I_{2Z}+2\pi J_{IS} I_1 \cdot I_2 \qquad \text{(S1)}$$

The *ω* represent the Larmor angular frequency, I_1 and I_2 represent density matrices of two scalar coupled nuclear spins in case of two coupled spins. The five ^{1}H and ^{19}F spins have a total of 5 Zeeman and 10 scalar coupling Hamiltonian terms.

Second, the effect of the pulses from pulse sequences were assumed to be instantaneous, which can be represented by *π or π/2* rotation matrices.

Third, all hydrogen atoms in the molecules were assumed to be 100% ^{1}H, and all interactions between ^{1}H and ^{19}F with ^{13}C, whose natural abundance of 1%, were ignored.

Dimensionless nuclear spin angular momentum of ½ spins, regardless of the proton and neutron numbers, can be represented by a matrix. Pauli matrices were used to represent the Cartesian unit directions.

$$\hat{I}_x=\frac{1}{2}\begin{bmatrix}0 & 1\\ 1 & 0\end{bmatrix},\hat{I}_y=\frac{1}{2}\begin{bmatrix}0 & -i\\ i & 0\end{bmatrix},\hat{I}_z=\frac{1}{2}\begin{bmatrix}1 & 0\\ 0 & -1\end{bmatrix} \quad \text{(S2)}$$

In a multispin system, the density matrix was described by the Kronecker product of matrices of individual spins. In both 3-fluoropyridine and 3,5-difluoropyridine, five magnetically nonequivalent ^{1}H and ^{19}F spins exist, necessitating a 32-by-32 density matrix.

$$\hat{I}=\hat{I}_1\otimes\hat{I}_2\otimes\hat{I}_3\otimes\hat{I}_4\otimes\hat{I}_5 \quad \text{(S3)}$$

The *I* denotes the density matrix, and the subscript denotes the specific ^{1}H or ^{19}F spin and its Cartesian direction. For a coherence with no relation to a specific spin, the corresponding Pauli matrix would be replaced by unit matrix *E*. For instance, the I_{3x} is derived as such.

$$\hat{I}_{3x}=\hat{E}\otimes\hat{E}\otimes\hat{I}_x\otimes\hat{E}\otimes\hat{E} \quad \text{(S4)}$$

The time evolution of the density matrix could be expressed by the Liouville-von Neumann equation,

$$\frac{d\hat{I}(t)}{dt}=i[\hat{I}(t),\hat{H}] \quad \text{(S5)}$$

The *t* denotes time. A solution exists for this complex differential equation.

$$\hat{R}(t)=e^{-i\hat{H}(t)} \quad \text{(S6)}$$

The *e* indicates a matrix exponential.

$$\hat{I}(t)=\hat{R}(t)\hat{I}(0)\hat{R}^{-1}(t) \quad \text{(S7)}$$

In the experiment, the NMR signal came from the induced electrical signal of *x* phase, meaning that the acquired signal should be the I_x component of the observed fluorine spins.

$$FID_{3-FPy}(t)=Tr(\hat{I}(t)\cdot\hat{I}_{3x}) \quad \text{(S8)}$$

$$FID_{3,5-diFPy}(t)=Tr(\hat{I}(t)\cdot\hat{I}_{3x})+Tr(\hat{I}(t)\cdot\hat{I}_{5x}) \quad \text{(S9)}$$

FID represents the acquired time domain signal, *Tr* represents the trace of the matrix. Subscript *3* indicates 3-F spin of 3-fluoropyridine and subscript *3,5* indicates 3- and 5-F spins of 3,5-difluoropyridine.

In the simulation, the density matrix evolution started with all spins aligned with I_z at equilibrium. The Hamiltonian was the sum of 5 free precession terms, corresponding to Larmor angular frequencies of each spin, and 10 scalar coupling terms from all combinations of two spins among the five. A density matrix was simulated for each direct-dimension spectrum to the end of the mixing pulse. For the time domain signal at acquisition, each data point was simulated

independently with identical parameters to the NMR experiment. The refocusing pulses were abstracted as instantaneous rotations that consumed zero time. The pulses matrices affected only ^{1}H and ^{19}F, consistent with the selective pulses used in the experiments. FID from phase cycled scans were combined, and the subsequent processing and analysis were identical to the experiment.

The ^{14}N with a spin quantum number of one is coupled to other nuclear spins. However, it possesses a much faster spin relaxation due to quadrupolar relaxation mechanism,[6] and its influence on the evolution of coherences of other spins is negligible and was therefore not considered in the simulations.[7]

1.5 High-Field NMR Experiments for Measurement of J-Coupling Constants

The ^{1}H and ^{19}F peaks of 3-fluoropyridine and 3,5-difluoropyridine were assigned by chemical shift. The ^{1}H-^{1}H homonuclear and ^{1}H-^{19}F heteronuclear scalar coupling constants of 3-fluoropyridine and 3,5-difluoropyridine were measured by ECOSY and IPAP-HSQMBC-TOCSY experiments.[8,9] The fluoropyridine samples were prepared by dissolving 50 mM of 3-fluoropyridine or 3,5-difluoropyridine (Ambeed Inc., Arlington Hts, IL) into fully deuterated methanol (Cambridge Isotope Laboratories, Inc., Andover, MA). The NMR spectra were measured on an Avance NEO 500 spectrometer with a BBF probe (Bruker Biospin, Billerica, MA). Hard pulses of ^{1}H and ^{19}F had γB_1 of 24.5 and 18.7 kHz, respectively. The 1D experiments had a spectral resolution of 0.867 Hz. The DIPSI-2 mixing pulses in IPAP-HSQMBC-TOCSY experiments were set to cover a span of 2 kHz with a duration of 13.8 ms, and the delays in the experiments were optimized to 20 Hz coupling, due to the presence of multiple ^{1}H to ^{19}F couplings.[9] The number of scans was 4 in IPAP experiments. ECOSY experiments used a number of scans of 2, 512 t_1 increments each of 0.667 ms. The spectral resolution in IPAP experiments was 0.090 Hz, and the resolutions in ECOSY experiments were 0.028 and 0.366 Hz for the direct and indirect axes respectively.

2. Low-field NMR Calibration Data

The bubble time was optimized though incrementation and a diminishing marginal maximum was found at 10 seconds, at bubble flow rate of 0.1 SLPM. 8 seconds bubbling as a trade-off for more scans was adopted for all SABRE experiments. The optimization of this bubbling time is shown in Figure S3.

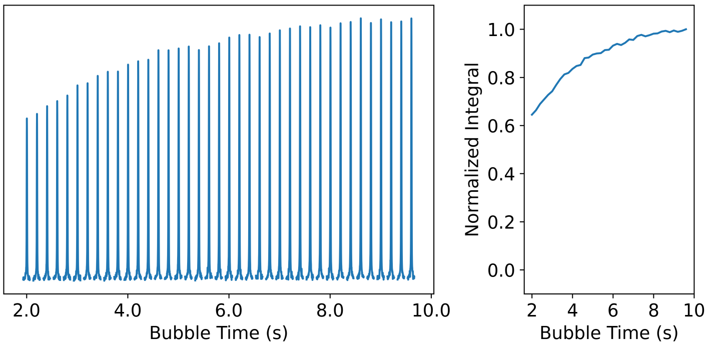

Figure S3: SABRE low-field NMR spectra (Left) and integral (Right) of ^{19}F peak by incremented parahydrogen bubbling time. The calibration experiment was performed at 0.82 mT.

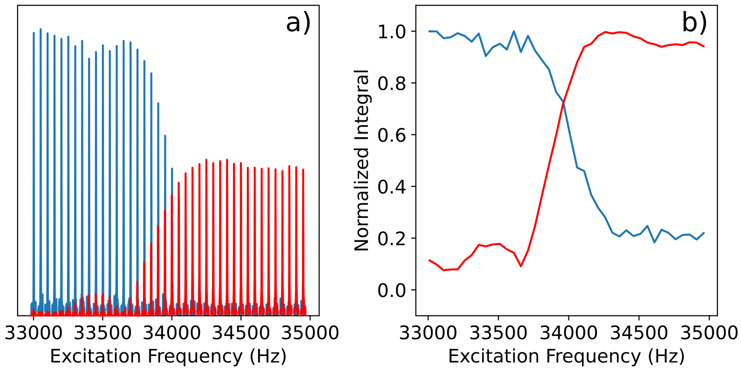

Figure S4: Excitation profile of a sinc-shaped pulse with a flip angle of nominally π/2, obtained by incrementing the pulse frequency from the Larmor frequency of ^{19}F to ^{1}H. a) Arrays of ^{19}F (blue) and ^{1}H (red) peaks plotted as slices extending from -300 to +300 Hz around the respective Larmor frequency. b) Integrals of the slices from -200 to +200 Hz around the Larmor frequency. The pulse envelope followed the sin(x)/x *function with x extending from -3π to 3π. The pulse had a length of 3.4 ms and amplitude of 0.35 V. The profile calibration experiment was SABRE hyperpolarized and performed at 0.82 mT.*

3. Density Matrix Simulation of 1D Experiments

The 1D CPMG spectra were simulated to confirm the modulated shape of the FID. The FID is further processed into spectra in the same way as 1D experiments to compare and buttress the results.

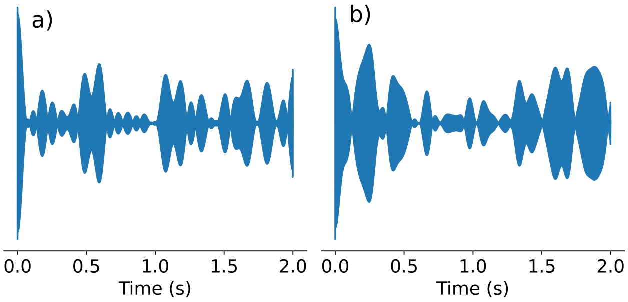

Figure S5: Simulated 1D time-resolved CPMG time-domain FID of a) 3-fluoropyridine and b) 3,5-difluoropyridine, in comparison to experimental FID in Figure 1. The simulation did not consider relaxation. The FID are processed the same way as the experiment FID, and the spectra are displayed as orange in Figure 1c and d. The simulations were performed at 0.82 mT.

4. Product Operator Description of 2D Experiments

The result of the pulse sequence in Figure 2 for a single ^{1}H-^{19}F spin pair is illustrated with a product operator calculation. This formalism does not extend to the strong coupling regime which many scalar couplings in the fluoropyridine molecules are in, and that the density matrix simulation scheme described below should be adopted. At the 0.82 mT field, the homonuclear couplings among ^{1}H and among ^{19}F spins are in the strong coupling regime, while the heteronuclear couplings are in the weak coupling regime.

Let $\hat{I}$ and $\hat{S}$ represent the density operator of ^{19}F and ^{1}H in a molecule that contained only one ^{19}F and ^{1}H spin each. The pulse sequence intends to select the t_1 dependent evolved anti-phase coherence, $\sin(\pi J t_1) 2 I_x S_z$, at the detection stage. The free evolution period produces a *z*-coherence of fixed sign and an anti-phase coherence of alternating sign.

$$\hat{I}_z \xrightarrow{\pm(\frac{\pi}{2}\hat{I}_x)} \mp\hat{I}_y \xrightarrow{(\pi\hat{I}_x+\pi\hat{S}_x)+2\pi J t_1} \cos(\pi J t_1)\hat{I}_z \mp \sin(\pi J t_1) 2\hat{I}_x\hat{S}_z \qquad \text{(S10)}$$

The two-step phase cycling excitation pulses removes the *z*-coherence and selects the anti-phase coherence, which evolves into observable in-phase coherence during the detection period, modulated by $\sin(2\pi J t_1)$. The refocusing pulses during the detection period does not alter the sign of both in-phase and anti-phase coherences, but only serves to produce echoes for observation.

$$\sin(\pi J t_1) 2\hat{I}_x \hat{S}_z \xrightarrow{\pm(\frac{\pi}{2}\hat{I}_x)} \xrightarrow{2\pi J t_1 + \Omega \hat{I}_z t_1} \tag{S11}$$

$$\sin(\pi J t_1)\cos(\Omega_I t_2)\cos(\pi J t_2) 2\hat{I}_x \hat{S}_z + \sin(\pi J t_1)\cos(\Omega_I t_2)\sin(\pi J t_2)\hat{I}_y$$
$$+\sin(\pi J t_1)\sin(\Omega_I t_2)\cos(\pi J t_2) 2\hat{I}_y \hat{S}_z - \sin(\pi J t_1)\sin(\Omega_I t_2)\sin(\pi J t_2)\hat{I}_x$$

$$-\sin(\pi J t_1)\sin(\Omega_I t_2)\sin(\pi J t_2)\hat{I}_x = \\ -\frac{1}{2}\sin(\pi J t_1)\cos((\Omega_I - \pi J)t_2)\hat{I}_x + \frac{1}{2}\sin(\pi J t_1)\cos((\Omega_I + \pi J)t_2)\hat{I}_x \tag{S12}$$

The observable in-phase coherences $\hat{I}_x$ create a doublet on the t_2 axis at $+J$ and $-J$ in respect to Larmor frequency, although the signs will not manifest in the magnitude spectrum. The doublet is positioned on the t_1 axis at $2J$ if a Fourier transform of absolute values of $sin(\pi J t_1)$ is applied for the indirect dimension.

The prediction of 2D spectrum peak pattern extends to multiple coupled spins system in the weak coupling regime. Let $\hat{I}$, $\hat{S}_1$ and $\hat{S}_2$ represent the density operator of one ^{19}F and two ^{1}H in a molecule respectively, and let J_1 and J_2 represent the coupling of $\hat{I}$ to $\hat{S}_1$ and $\hat{S}_2$ respectively, and all other couplings ignored. The final mixing pulse selects the two anti-phase coherences for detection, while the *z*- and triple quantum coherences are eliminated by subtraction of FID in phase cycles.

$$\hat{I}_z \xrightarrow{\pm(\frac{\pi}{2}\hat{I}_x)} \xrightarrow{(\pi \hat{I}_x + \pi \hat{S}_x) + 2\pi J t_1} \xrightarrow{\pm(\frac{\pi}{2}\hat{I}_x)} \tag{S13}$$

$$\mp\sin(\pi J_1 t_1)\cos(\pi J_2 t_1) 2\hat{I}_x \hat{S}_{1z} \mp \sin(\pi J_2 t_1)\cos(\pi J_1 t_1) 2\hat{I}_x \hat{S}_{2z}$$
$$+\cos(\pi J_1 t_1)\cos(\pi J_2 t_1)\hat{I}_z + \sin(\pi J_1 t_1)\sin(\pi J_2 t_1) 4\hat{I}_z \hat{S}_{1z}\hat{S}_{2z}$$

During the detection, all other anti-phase, multiple quantum coherences and *y*-phased in-phase coherences are not observable and therefore are ignored.

$$\sin(\pi J_1 t_1)\cos(\pi J_2 t_1) 2\hat{I}_x \hat{S}_{1z} + \sin(\pi J_2 t_1)\cos(\pi J_1 t_1) 2\hat{I}_x \hat{S}_{2z} \xrightarrow{2\pi J t_1 + \Omega \hat{I}_z t_1}$$
$$\sin(\pi J_1 t_1)\cos(\pi J_2 t_1)\sin(\Omega_I t_2)\sin(\pi J_1 t_2)\cos(\pi J_2 t_2)\hat{I}_x$$
$$+\sin(\pi J_2 t_1)\cos(\pi J_1 t_1)\sin(\Omega_I t_2)\sin(\pi J_2 t_2)\cos(\pi J_1 t_2)\hat{I}_x \tag{S14}$$
$$= \frac{1}{4}\sin(\pi(J_1+J_2)t_1)[\cos((\Omega_I + \pi J_1 + \pi J_2)t_2) - \cos((\Omega_I - \pi J_1 - \pi J_2)t_2)]\hat{I}_x$$
$$+\frac{1}{4}\sin(\pi(J_1-J_2)t_1)[\cos((\Omega_I - \pi J_1 + \pi J_2)t_2) - \cos((\Omega_I + \pi J_1 - \pi J_2)t_2)]\hat{I}_x$$

The 8 terms each from one anti-phase coherence add constructively and destructively at different places with those from the other, and lead to 4 observable peaks arranged in a “V” shape in total (*Figure S6*). Two peaks are at $2(J_1+J_2)$ on the indirect axis and at J_1+J_2 and $-J_1-J_2$ with respect to Larmor frequency on the direct axis. The other two peaks are at $2|(J_1-J_2)|$ on the indirect axis and at J_1-J_2 and $-J_1+J_2$ with respect to Larmor frequency on the direct axis, all on the two “diagonal” lines drawn from the origin point with slopes of 2 and -2. The factor 2 of indirect axis positions is coming from the real Fourier transform of absolute values of sine functions for the indirect axis, while

noises at zero and harmonics of the proper frequencies are expected. Additional couplings among ^{1}H spins and among ^{19}F spins fall in the strong coupling regime and would break the "V" shape discussed above, and could be studied through the density matrix simulation.

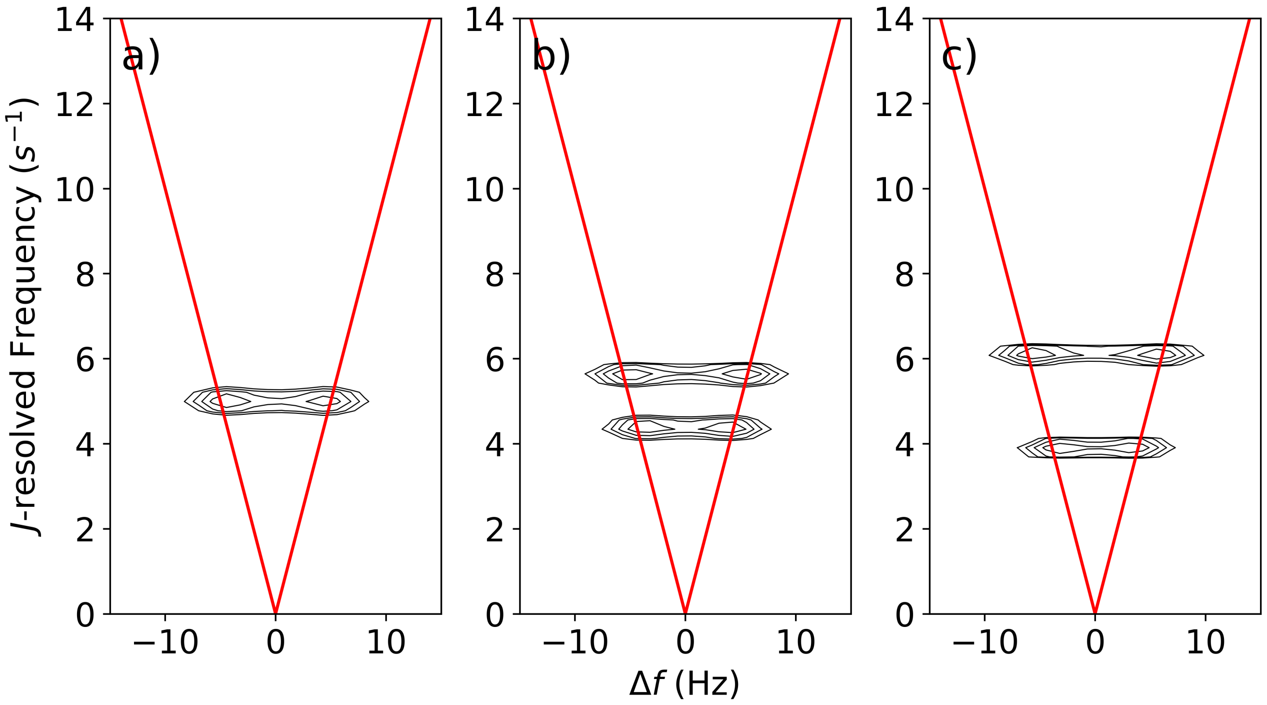

Figure S6: Simulated spectra for a three-spin system (I, S1, S2) at 0.85 mT as shown in Figure 5 in the main text, where J_{IS1}*= 5 Hz,* J_{S1S2} *= 0 and a)* J_{IS2} *= 0, b)* J_{IS2} *= 0.5 and c)* J_{IS2} *= 1 Hz. The signal of the I spin is shown. The red "V" shape is predicted by product operators. Other than in Figure 5, the spectra were not processed by calculating the magnitude.*

5. Validation of 2D Spectra

In presence of molecular diffusion and B_0 inhomogeneity, the relaxation is expected to be slower with a shorter inter-pulse delay, resulting in less signal loss.[10] The effect of shorter inter-pulse delay was tested by using five refocusing pulses instead of one in the longest evolution time period (*Figure S7*). With more refocusing pulses, the integrated signal intensity decreased, suggesting that the improvement over the loss of signal was outweighed by the cumulative imperfection of the refocusing pulses.

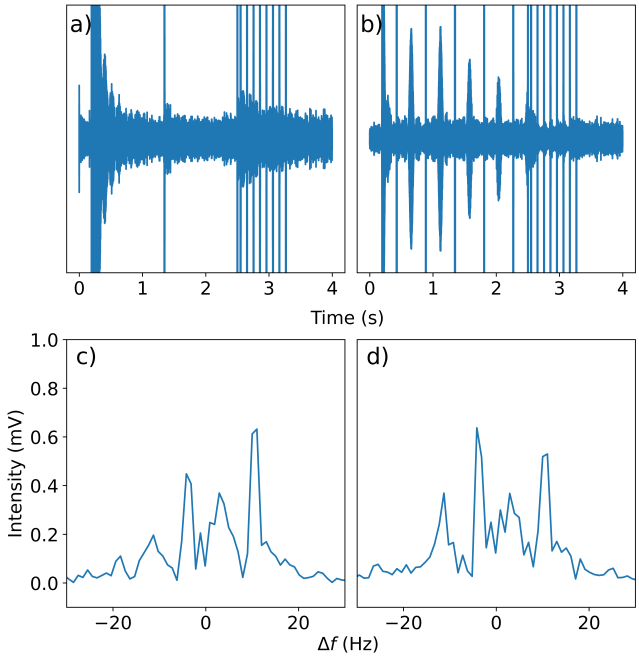

Figure S7: Comparison of the experiment with a single vs. multiple evenly-timed refocusing pulses applied during evolution. a) The FID of the longest t_1 *time slice in Figure 3. b) The FID with five refocusing pulses during* t_1 *time, with same parameters as a). c) The processed 1D spectrum from a). d) The processed spectrum from b). The experiments were performed at 0.82 mT with SABRE hyperpolarization.*

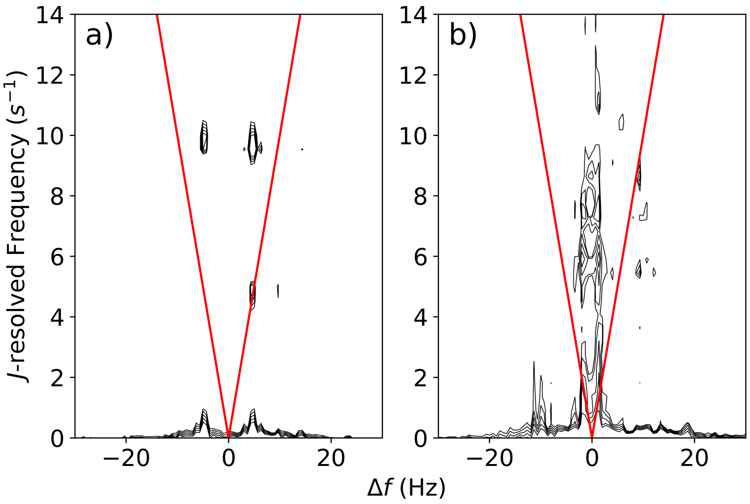

Figure S8: A repetition of Figure 4. Two-dimensional J-resolved ^{19}F spectra contour plot in logarithmic scale of 3,5-difluoropyridine (a) and 3-fluoropyridine (b) from the experiment, respectively. The direct axis represent the frequency of the signal, and the indirect axis is interpreted to originate from the coupling evolution from the free evolution time. The experiments were performed at 0.82 mT with SABRE hyperpolarization.

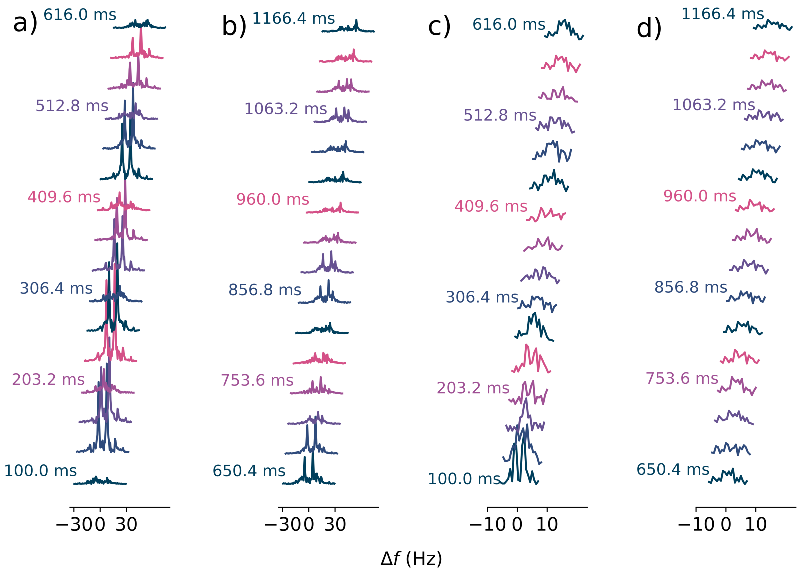

Figure S9: Stack plots of 1D ^{19}F spectra of the 2D J-resolved experiments on slice 1-16 (a) and 17-32 (b) and of 3,5-difluoropyridine and slice 1-16 (c) and 17-32 (d) of 3-fluoropyridine. The evolution time are marked to the corresponding slices. The experiments were performed at 0.82 mT with SABRE hyperpolarization.

6. Density Matrix Simulation of 2D Experiments

The simulated spectra were evaluated and matched manually on the positions and relative intensities of all major peaks, with a priority on positions because of the clearer defined frequencies being less prone to experimental imperfections than intensities. The values of coupling constants were assumed to generally adhere to the experimental values, and as such, the signs of the coupling constants were optimized first by trying all combinations. The values were matched through alternating variations on all coupling constants, one at a time and in a back-and-forth fashion, prioritizing the couplings involving ^{19}F and with higher absolute values.

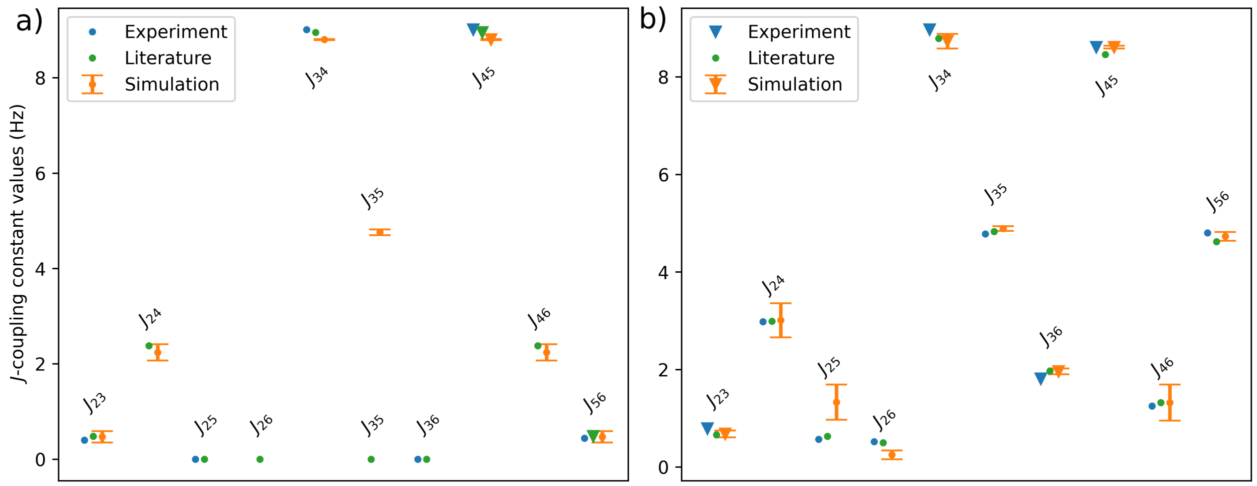

Figure S10: The magnitudes of coupling constants between ^{1}H and ^{19}F spins on a) 3,5-difluoropyridine and b) 3-fluoropyridine (FPy) measured with high-field multidimensional NMR experiments (Experiment), simulated using density matrix (Simulation), compared to signed reported values (Literature)[6,11], in Hz. The positive signs are marked by dots, and negative by triangles. The J_{35} ^{1}H-^{1}H coupling have different simulation and literature values and are both labeled. The data are listed Table S1.

Table S1: The coupling constants between ^{1}H and ^{19}F spins on 3,5-difluoropyridine (diFPy) and 3-fluoropyridine (FPy) measured with high-field multidimensional NMR experiments (exp), simulated using density matrix (sim), compared to signed reported values (lit),[6,11] in Hz. In experiment and simulation results, the blanks represent those that cannot be resolved and "0" represents those determined to be zero. The errors were determined by positional partial derivative and the spectra resolution. The results are visualized in Figure S10.

	J_{23}	J_{24}	J_{25}	J_{26}	J_{34}	J_{35}	J_{36}	J_{45}	J_{46}	J_{56}
diFPy, exp	0.44		0		9.01		0	-9.01		0.44
diFPy, lit (sim)	0.48	2.38	0	0	8.95	0	0	-8.95	2.38	-0.48
diFPy, sim	0.47 ± 0.12	2.24 ± 0.17			8.80 ± 0.01	4.76 ± 0.06		-8.80 ± 0.01	2.24 ± 0.17	0.47 ± 0.12
FPy, exp	-0.79	2.98	0.57	0.52	-8.97	4.78	-1.81	-8.61	1.25	4.80
FPy, lit (exp)	0.66	2.99	0.63	0.5	8.79	4.83	1.97	8.46	1.32	4.62
FPy, sim	-0.68 ± 0.07	3.01 ± 0.35	1.33 ± 0.26	0.25 ± 0.09	-8.73 ± 0.15	4.89 ± 0.05	-1.96 ± 0.06	-8.61 ± 0.03	1.32 ± 0.37	4.73 ± 0.09

The error estimation (*Figure S11*) of the resulting coupling constants were evaluated by incrementing one coupling constant, or a pair of symmetrical coupling constants, and was calculated by the division of spectral resolution by the *x* and *y* partial derivative of all peaks' positions to the *J*, as the simulation was seen as closest match whose peak positions were exact and contributed to the error by one unit of spectra resolution on *x* and *y* separately. The overall error is calculated from the propagation of *x* and *y* errors.

$$Err_{x,y} = |SR_{x,y}| \frac{dJ}{d\delta_{x,y}} \tag{S15}$$

$$Err = \sqrt{Err_x^2 \delta_x^2 + Err_y^2 \delta_y^2} \tag{S16}$$

The *Err* represents error, *SR* represents the spectral resolution, δ represents the frequency offset on the 2D spectrum and *J* is the coupling constant.

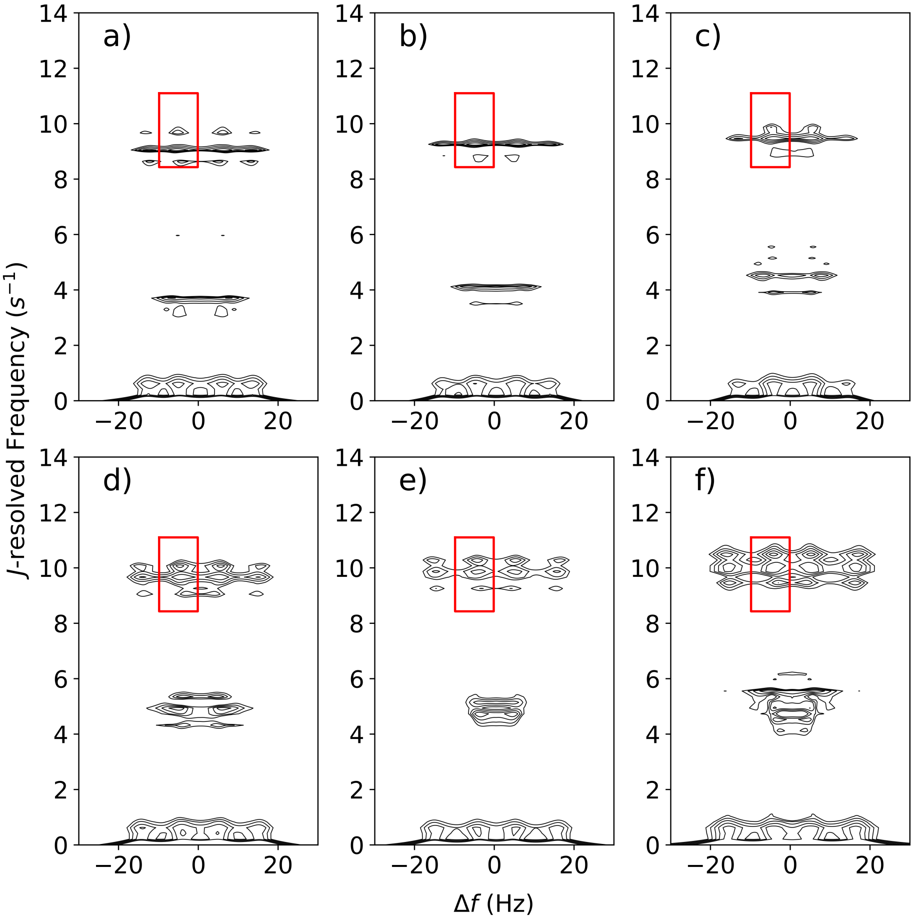

Figure S11: Simulations of incremented ^{19}F-^{19}F coupling constant on 3,5-difluoropyridine. The coupling constants were a) 3.99, b) 4.38, c) 4.76, d) 5.15, e) 5.53 and f) 5.92. The red box indicates the positions range of peaks with the most prominent intensity and changes. The simulations were performed at 0.82 mT.

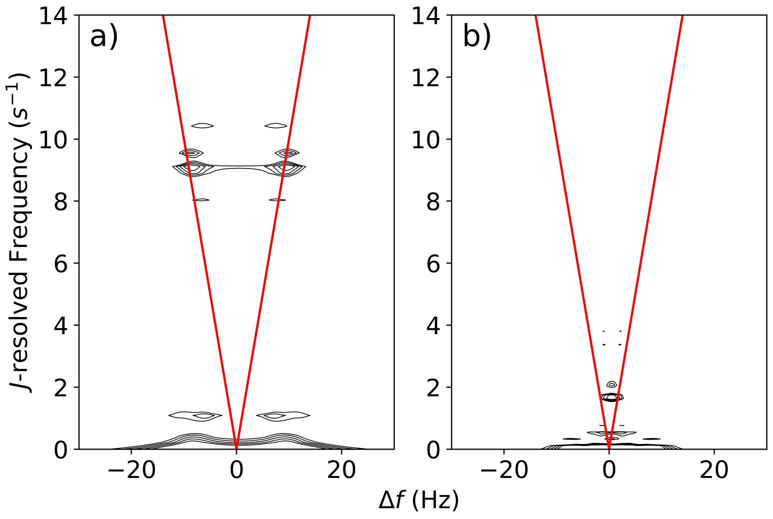

Figure S12: *Simulated 2D ^{19}F spectra contour plot in logarithmic scale* of *3,5-difluoropyridine (Left) and 3-fluoropyridine (Right), based on reported coupling constants, respectively.*[6,11] *The simulations were performed at 0.82 mT.*

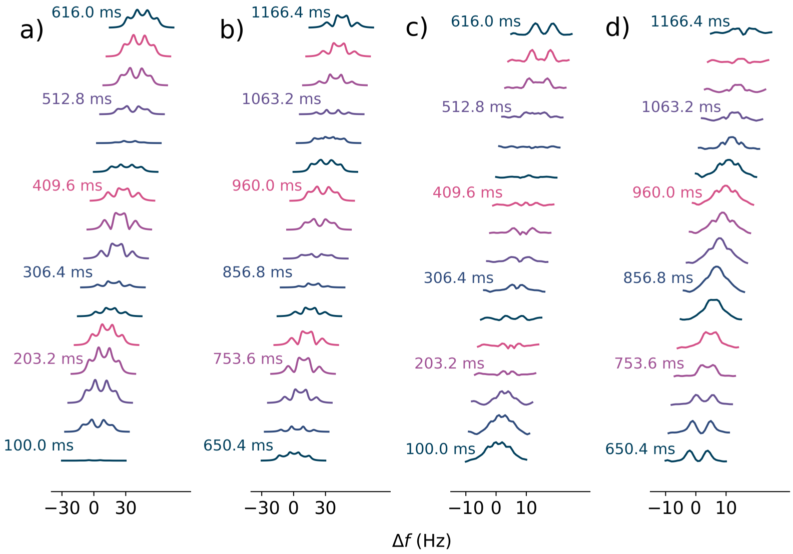

Figure S13: Stack plots of 1D ^{19}F spectra of the 2D J-resolved simulation on slice 1-16 (a) and 17-32 (b) and of 3,5-difluoropyridine and slice 1-16 (c) and 17-32 (d) of 3-fluoropyridine. The evolution times are marked to the corresponding slices. The simulations were performed at 0.82 mT.

7. Measurement of Scalar Coupling Constants by High-Field NMR

Chemical shifts of ^{1}H and ^{19}F spins are assigned in 1D spectra (*Figure S14*).

In the ECOSY experiment, the ^{1}H-^{1}H homonuclear coupling constants values are determined by measuring the horizontal and vertical distance of square-connected multiple peaks, and the distance of square-connected multiple peaks splittings. The number of squares is equal to 2^{n-2} where *n* is the number of non-equivalent homonuclear spins and must be equal or greater than 3, making ^{1}H homonuclear couplings in 3,5-difluoropyridine unobservable (*Figure S15*). The four ^{1}H spins in 3-fluoropyridine make four square-connected multiple peaks, shifting in a parallelogram style. The two adjacent sides of the parallelogram can interpret 4 coupling constants. The identity of one coupled-spin is the spin represented by the chemical shift assignment from horizontal/vertical axis, depending on the horizontal/vertical frequency shift one is referring to. The identity of the other spin is inferred from other spectra so all agrees. The signs of the coupling constant are assigned relatively, so the horizontal and vertical frequency shift make a product greater than zero when shift is top-right/ bottom-left tilt, and smaller than zero when top-left/bottom-right tilt.[8] In Figure S16a, at the frequency of 5-H (indirect) and 6-H (direct), the coupling of a spin causes shift in 8.61 Hz on indirect axis and 1.25 Hz on direct axis. The value matches with reported 4-H couplings to 5- and 6-H. The top-left/bottom-right tilt indicates opposite signs between these two values. In Figure S16b, at the frequency of 5-H (indirect) and 4-H (direct), the coupling of a spin causes shift in 4.80 Hz on indirect axis and 1.25 Hz on direct axis. The value matches with reported 6-H couplings to 5- and 4-H. The top-right/bottom-left tilt indicates same signs between these two values. The absolute signs of all ^{1}H homonuclear couplings are assigned to give least deviation from the literature values.[11]

In the IPAP-HSQMBC-TOCSY the ^{1}H-^{19}F heteronuclear coupling constants are determined by the directional frequency offset between the IP-AP and IP+AP spectra (*Figure S17-18*). The IP-AP shifting to right from IP+AP spectra indicates positive sign, and the shift is interpreted as the value of the coupling constant.

These experiments determine the signs and values of the ^{1}H-^{19}F and ^{1}H-^{1}H coupling constants, respectively, and yielded the a combination of positive and negative values.[11] Large coupling constant values of as high as 9.01 Hz were found for ^{1}H-^{19}F couplings in 3,5-difluoropyridine and 3-fluoropyridine. The coupling constants of 3,5-difluoropyridine were found to be anti-symmetrical. Both ^{1}H-^{1}H homonuclear and ^{1}H-^{19}F heteronuclear coupling were found to range between 9 and 0 for 3-fluoropyridine, and couplings involving both 3-F, 4-H and 5-H were found to be generally higher than those involving one or none. ECOSY spectrum of 3,5-difluoropyridine was not able to resolve any coupling because of its lack of a third nonequivalent ^{1}H spin. The measured coupling constant values of 3,5-difluoropyridine matched those in a previous simulation The anti-symmetrical signs of coupling constants of symmetrical spins on 3,5-difluoropyridine were attributed to the anti-symmetry of the bonding electron orbitals. The strongest couplings of both molecules were found to be J_{34} and J_{45}, implying the underlying pyridine delocalized electron orbital configuration was only weakly influenced by fluorine substitutions. The anti-symmetrical *J*-couplings are similar to the anti-symmetrical HOMO orbitals of pentafluoropyridine.[12]

The coupling constant of the molecule in deuterated chloroform and trifluoroacetic acid differ, therefore a similar minor disagreement would be expected for the methanol solvent used in this study.

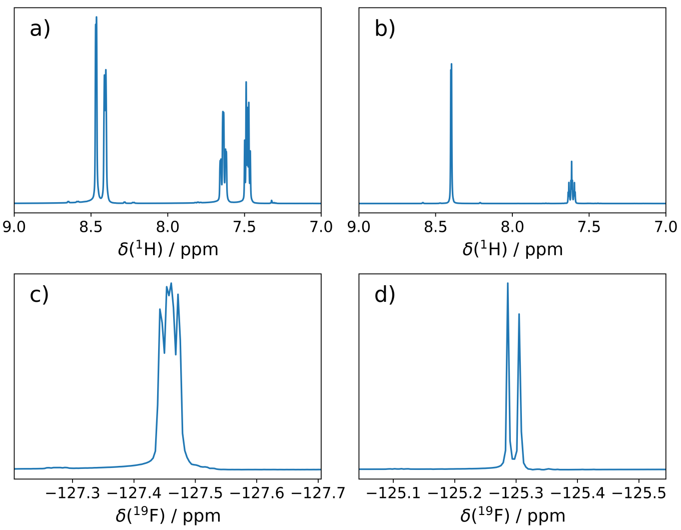

Figure S14: The ^{1}H spectra of a) 3-fluoropyridine and b) 3,5-difluoropyridine. The chemical shifts of 2-, 4-, 5- and 6-H of 3-fluoropyridine are assigned to 8.467, 7.638, 7.480 and 8.407 ppm respectively. The chemical shifts of 2-/6-H and 4-H of 3,5-difluoropyridine are assigned to 8.399 and 7.614 ppm respectively. The ^{19}F spectra of c) 3-fluoropyridine and d) 3,5-difluoropyridine. the chemical shift of 3-F of 3-fluoropyridine is assigned to -127.456 ppm and the chemical shifts of 3- and 5-F of 3,5-difluoropyridine are assigned to -125.295 ppm.

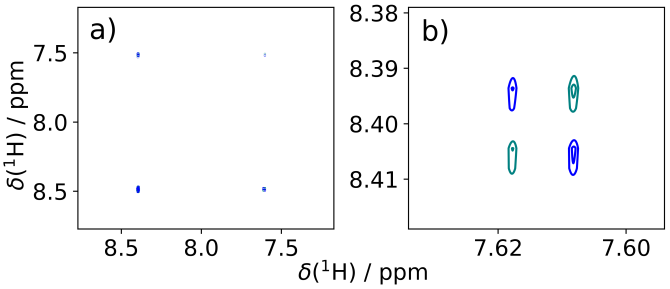

Figure S15: The a) ECOSY spectra of 3,5-difluoropyridine, and the b) ortho-para ^{1}H cross peaks. The blue contour represents positive peaks, and green contour represents negative peaks. The spectra are reported in logarithm scale. The absence of shift of square denotes absence of three non-equivalent ^{1}H spins.

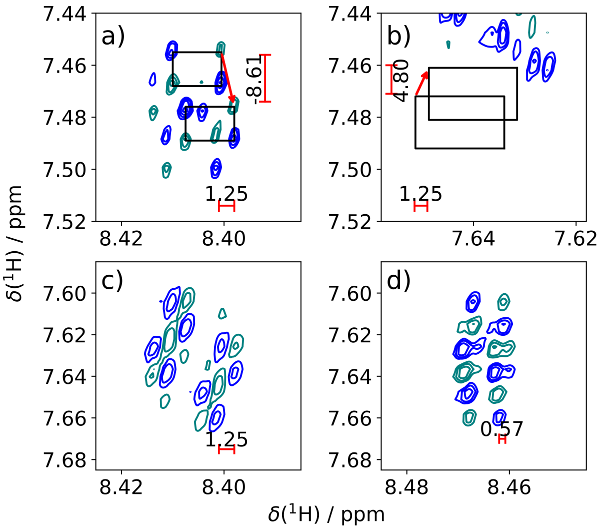

Figure S16: The ECOSY spectra of 3-fluoropyridine showing the cross peaks of a) 5-6, b) 5-4, c) 4-6, d) 4-2 ^{1}H-^{1}H spins. The blue contour represents positive peaks, and green contour represents negative peaks. The spectra are reported in logarithm scale. The square cross peak patterns in a) shift caused by J_{45} = -8.61 Hz and J_{46} = 1.25 Hz couplings and in b) caused by J_{56} = 4.80 Hz and J_{46} = 1.25 Hz couplings are marked by black boxes, showing relative signs through tilt angle. The values measured are annotated to the side.

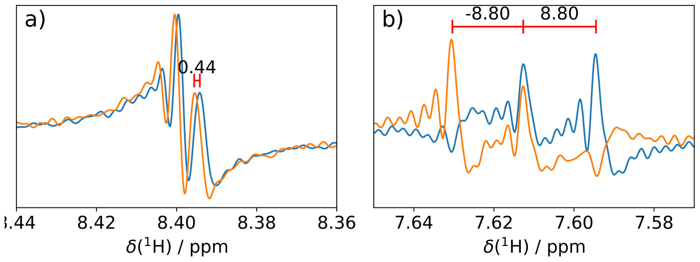

Figure S17: The IPAP-HSQMBC-TOCSY spectra of 3,5-difluoropyridine on the cross peaks of a) ortho- and b) para-^{1}H to meta-^{19}F spins. The orange curve represents IP+AP spectra, and blue curve represents IP-AP spectra.

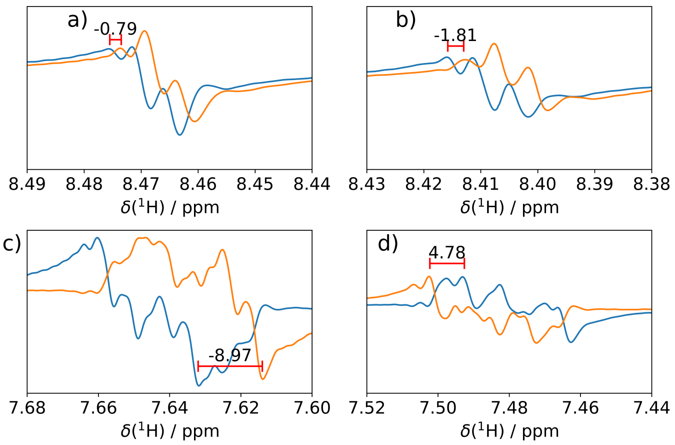

Figure S18: The IPAP-HSQMBC-TOCSY spectra of 3-fluoropyridine of a) 2-, b) 6-, c) 4- and d) 5- ^{1}H to 3-^{19}F spins. The orange curve represents IP+AP spectra, and blue curve represents IP-AP spectra.

8. Simulation of Molecules with Expanded Scope

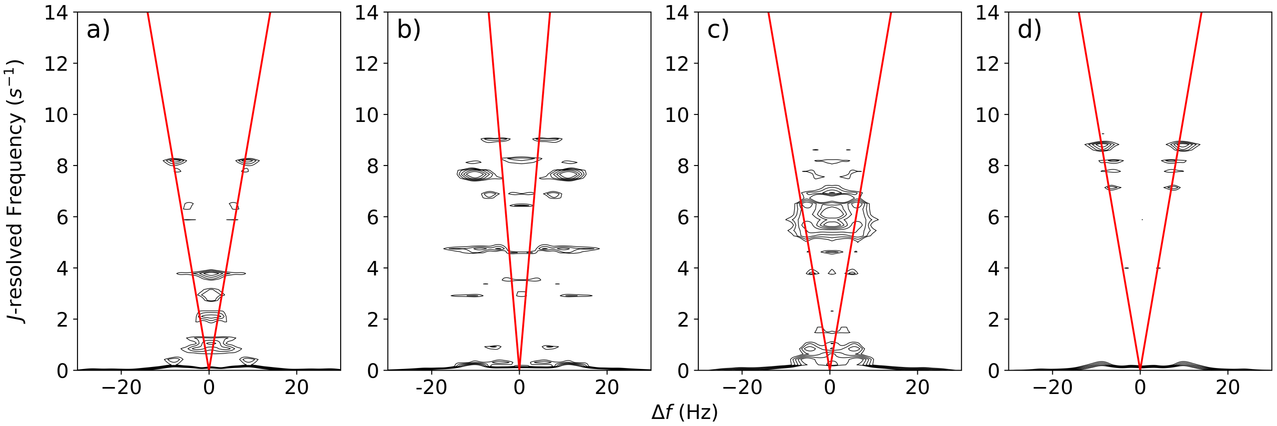

Figure S19: Simulated 2D ^{19}F spectra in logarithmic scale of a) 2-fluoropyridine, b) 2,3-difluoropyridine, c) 2,5-difluoropyridine and d) 2,6-difluoropyridine. The ^{1}H and ^{19}F spins were simulated. The t_1 *increment was 0.0344 s.*[11] *The simulations were performed at 0.82 mT.*

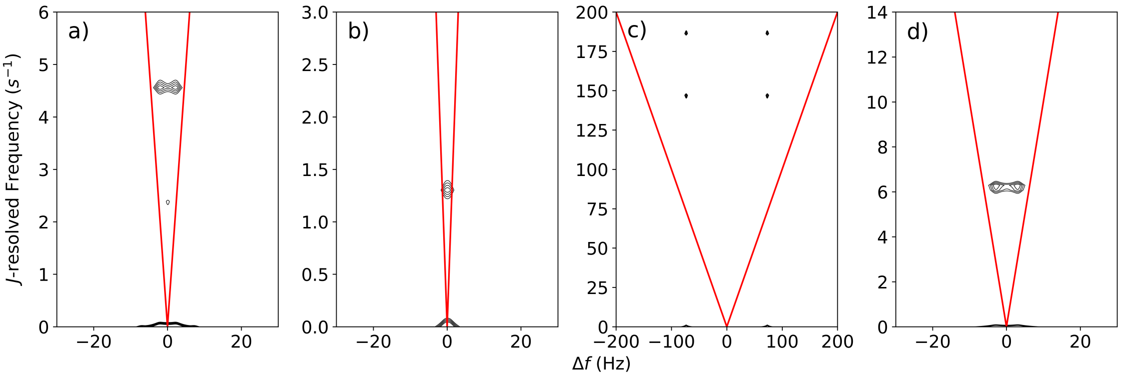

Figure S20: Simulated 2D ^{13}C spectra in logarithmic scale of a) [1-^{13}C] lactic acid, b) [1-^{13}C] pyruvic acid, c) [2-^{13}C] lactic acid, d) [2-^{13}C] pyruvic acid. The ^{1}H-^{13}C and ^{1}H-^{1}H coupling constants were based on reported values.[13,14] *The ^{1}H and ^{13}C spins were simulated at 0.82 mT. The* t_1 *increment was 0.009 s except for [2-^{13}C] lactic acid, where it was 0.0015 s. Note the different axis in (c) due to the 146.3 Hz ^{1}H-^{13}C single bond coupling in [2-^{13}C] lactic acid.*

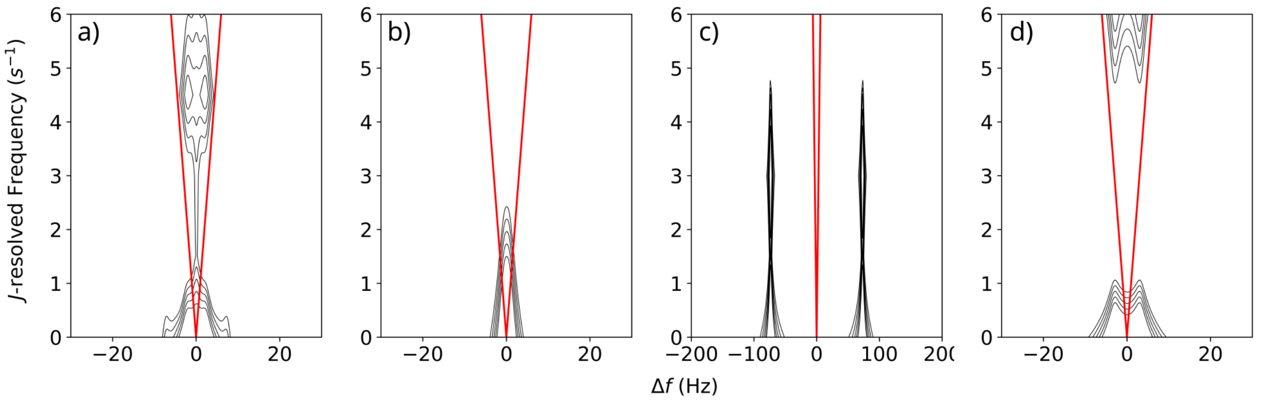

Figure S21: Simulated fast 2D ^{13}C spectra contour plot in logarithmic scale of (a) [1-^{13}C] lactic acid, (b) [1-^{13}C] pyruvic acid, (c) [2-^{13}C] lactic acid, (d) [2-^{13}C] pyruvic acid. The ^{1}H-^{13}C and ^{1}H-^{1}H coupling constants were based on the same reported values as Figure S21. The ^{1}H and ^{13}C spins were simulated. The evolution time increment was 0.04167 s, and only 8 t_1 *increments were used for all simulations. The evolution time and* t_1 *increments are chosen specifically to quickly distinguish these four molecules. The simulations were performed at 0.82 mT. Note the [2-^{13}C] pyruvic acid does not have observable signal at low frequency.*

9. References

(1) Zhu, Y.; Chen, C.-H.; Wilson, Z.; Savukov, I.; Hilty, C. Milli-Tesla NMR and Spectrophotometry of Liquids Hyperpolarized by Dissolution Dynamic Nuclear Polarization. *J. Magn. Reson.* **2016**, *270*, 71–76. https://doi.org/10.1016/j.jmr.2016.06.014.

(2) Zhu, Y.; Hilty, C.; Savukov, I. Dynamic Nuclear Polarization Enhanced Nuclear Spin Optical Rotation. *Angew. Chem.* **2021**, *60* (16), 8823–8826. https://doi.org/10.1002/ange.202016412.

(3) Gottardi, G.; Mesirca, P.; Agostini, C.; Remondini, D.; Bersani, F. A Four Coil Exposure System (Tetracoil) Producing a Highly Uniform Magnetic Field. *Bioelectromagnetics* **2003**, *24* (2), 125–133. https://doi.org/10.1002/bem.10074.

(4) Meiboom, S.; Gill, D. Modified Spin-Echo Method for Measuring Nuclear Relaxation Times. *Rev. Sci. Instrum.* **1958**, *29* (8), 688–691. https://doi.org/10.1063/1.1716296.

(5) Hosur, R. V.; Kakita, V. M. R. *A Graduate Course in NMR Spectroscopy*; Springer International Publishing: Cham, 2022. https://doi.org/10.1007/978-3-030-88769-8.

(6) Altenhof, A. R.; Kaseman, D. C.; Mason, H. E.; Alvarez, M. A.; Malone, M. W.; Williams, R. F. On the Effects of Quadrupolar Relaxation in Earth's Field NMR Spectra. *J. Magn. Reson.* **2023**, *355*, 107540. https://doi.org/10.1016/j.jmr.2023.107540.

(7) Tatman, B. P.; Modha, H.; Brown, S. P. Comparison of Methods for ^{14}N-^{1}H Recoupling in ^{14}N-^{1}H HMQC MAS NMR. *J. Magn. Reson.* **2023**, *352*, 107459. https://doi.org/10.1016/j.jmr.2023.107459.

(8) Griesinger, C.; Sørensen, O. W.; Ernst, R. R. Practical Aspects of the E.COSY Technique. Measurement of Scalar Spin-Spin Coupling Constants in Peptides. *J. Magn. Reson. 1969* **1987**, *75* (3), 474–492. https://doi.org/10.1016/0022-2364(87)90102-8.

(9) Saurí, J.; Espinosa, J. F.; Parella, T. A Definitive NMR Solution for a Simple and Accurate Measurement of the Magnitude and the Sign of Small Heteronuclear Coupling Constants on Protonated and Non-Protonated Carbon Atoms. *Angew. Chem. Int. Ed.* **2012**, *51* (16), 3919–3922. https://doi.org/10.1002/anie.201108959.

(10) Carr, H. Y.; Purcell, E. M. Effects of Diffusion on Free Precession in Nuclear Magnetic Resonance Experiments. *Phys. Rev.* **1954**, *94* (3), 630–638. https://doi.org/10.1103/PhysRev.94.630.

(11) Thomas, W. A.; Griffin, G. E. The NMR Spectra of Some Fluorinated Pyridine Derivatives. *Org. Magn. Reson.* **1970**, *2* (5), 503–510. https://doi.org/10.1002/mrc.1270020507.

(12) Fuhrer, T. J.; Houck, M.; Iacono, S. T. Fluoromaticity: The Molecular Orbital Contributions of Fluorine Substituents to the π-Systems of Aromatic Rings. *ACS Omega* **2021**, *6* (48), 32607–32617. https://doi.org/10.1021/acsomega.1c04175.

(13) Marjańska, M.; Iltis, I.; Shestov, A. A.; Deelchand, D. K.; Nelson, C.; Uğurbil, K.; Henry, P.-G. *In Vivo* 13C Spectroscopy in the Rat Brain Using Hyperpolarized [1-13C]Pyruvate and [2-13C]Pyruvate. *J. Magn. Reson.* **2010**, *206* (2), 210–218. https://doi.org/10.1016/j.jmr.2010.07.006.

(14) Rodriguez, G. G.; von Petersdorff-Campen, C.; Korchak, S.; Sucre, O.; Santi, M. D.; Elsasser, J.; Mei, R.; Fries, L. M.; Felger, J.; Markus, A.; Alves, F.; Glöggler, S. Biological J-Coupling Spectroscopy at Low Magnetic Field. *Small Sci.* **2025**, *5* (11), 2500268. https://doi.org/10.1002/smsc.202500268.
